# Spatial-temporal heterogeneity and meteorological factors of hand-foot-and-mouth disease in Xinjiang, China from 2008 to 2016

**DOI:** 10.1371/journal.pone.0255222

**Published:** 2021-08-02

**Authors:** Ling Xie, Ruifang Huang, Hongwei Wang, Suhong Liu

**Affiliations:** 1 College of Resources and Environmental Sciences, Xinjiang University, Urumqi, China; 2 School of Environment and Resources, Guangxi Normal University, Guilin, China; 3 Center for Disease Control and Prevention of Xinjiang Uygur Autonomous Region, Urumqi, China; 4 Faculty of Geographical Science, Beijing Normal University, Beijing, China; GGS, UNITED STATES

## Abstract

The study aims to depict the temporal and spatial distributions of hand-foot-and-mouth disease (HFMD) in Xinjiang, China and reveal the relationships between the incidence of HFMD and meteorological factors in Xinjiang. With the national surveillance data of HFMD in Xinjiang and meteorological parameters in the study area from 2008 to 2016, in GeoDetector Model, we examined the effects of meteorological factors on the incidence of HFMD in Xinjiang, China, tested the spatial-temporal heterogeneity of HFMD risk, and explored the temporal-spatial patterns of HFMD through the spatial autocorrelation analysis. From 2008 to 2016, the HFMD distribution showed a distinct seasonal pattern and HFMD cases typically occurred from May to July and peaked in June in Xinjiang. Relative humidity, precipitation, barometric pressure and temperature had the more significant influences on the incidence of HFMD than other meteorological factors with the explanatory power of 0.30, 0.29, 0.29 and 0.21 (P<0.000). The interaction between any two meteorological factors had a nonlinear enhancement effect on the risk of HFMD. The relative risk in Northern Xinjiang was higher than that in Southern Xinjiang. Global spatial autocorrelation analysis results indicated a fluctuating trend over these years: the positive spatial dependency on the incidence of HFMD in 2008, 2010, 2012, 2014 and 2015, the negative spatial autocorrelation in 2009 and a random distribution pattern in 2011, 2013 and 2016. Our findings revealed the correlation between meteorological factors and the incidence of HFMD in Xinjiang. The correlation showed obvious spatiotemporal heterogeneity. The study provides the basis for the government to control HFMD based on meteorological information. The risk of HFMD can be predicted with appropriate meteorological factors for HFMD prevention and control.

## Introduction

Hand-foot-and-mouth disease (HFMD) is an infectious disease related to various enteroviruses that mostly affect children aged below 5 [1]. Its pathogens are typically coxsackieviruses (coxsackievirus A16 (CVA16)) and enteroviruses (enterovirus 71 (EV71)) [2–5]. Its clinical manifestations mainly include mouth ulcers, fever, and vesicles on the hands, feet, and mouth [6]. Most HFMD patients can fully recover since HFMD is a self-limited disease, but some patients may develop severe life-threatening complications and even death [3].

In recent years, HFMD outbreaks have been reported frequently in Asian countries such as Vietnam, Thailand, Singapore, Malaysia and China [7,8]. In 2014, China had large-scale outbreaks of HFMD and the cumulative cases reached 2,778,861 [9]. In May 2008, HFMD was added into Category C of notifiable diseases for disease surveillance in China [10]. Thus, HFMD is increasingly widely concerned.

The correlations between the incidence of HFMD and various meteorological factors such as relative humidity [1,11,12], precipitation [12] and temperature [12,13] have been extensively explored. The incidence of HFMD showed different characteristics in various countries and regions with distinct climate conditions [14]. In Vietnam [15], when monthly average air temperature above 26 °C increased by every 1 °C and monthly average relative humidity above 76% increased by every 1%, the incidence of HFMD respectively increased by 7% (RR:1.07; 95%CI: 1.052–1.088) and 3.1% (RR: 1.031, 95%CI: 1.024–1.039). However, the incidence of HFMD decreased by 3.1% when monthly average precipitation increased by every 1 mm. In South Korea [16], when monthly average air temperature below 18°C increased by every 1°C, the incidence of HFMD increased by 10.3% (95% CI: 8.4, 12.3%). In Sichuan, China [17], when monthly average relative humidity under 65% increased by every 1%, the incidence of HFMD increased by 6.6% (95% CI: 3.6, 9.7%); when monthly average relative humidity above 65% increased by every 1%, the incidence of HFMD decreased by 1.5% (95% CI: 0.4, 2.7%). The annual incidence of HFMD was positively correlated with annual average air temperature (RR: 1.171, 95% CI: 1.0435–1.3134).

In previous studies, different methods were adopted to explore the relationships between the incidence of HFMD and meteorological factors in four aspects. Firstly, the mathematical model was used to predict HFMD, such as seasonal auto-regressive integrated moving average (SARIMA) models [3] and SIR model [18]. Secondly, the changes in meteorological factors were used to estimate the risk of HFMD with regression models, such as time-series Poisson regression models [5]. Thirdly, time lag of HFMD and changes in meteorological factors were explored with the generalized additive model [19], the negative binomial multivariable regression model [20], and the distributed lag non-linear model [21]. Fourthly, the driving factors of HFMD were investigated with spatial panel data models [22] and GeoDetector [14]. The geographical detector is an appropriate model for quantifying the determinant power of driving factors of HFMD in Xinjiang. It is a set of statistical methods for detecting spatial differentiation and revealing the driving forces behind it.

To sum up, most of previous studies were mostly concentrated in areas with high incidence of HFMD, such as Hongkong [20], Guangdong [13], Guangxi [23] and Jiangsu [24]. The climate in these areas is significantly different from that in arid and semi-arid areas in Northwest China. The HFMD cases ranked first in Category C infectious diseases in Xinjiang in 2019 [25] and the number of HFMD cases increased gradually year by year. However, the correlations between HFMD and meteorological factors in Xinjiang have not been proved. In this study, we analyzed the spatial-temporal heterogeneity of HFMD and its relationship with meteorological factors in Xinjiang at the county scale in order to reveal the transmission mechanisms of HFMD in these semi-arid regions. The study aims to explore the spatiotemporal distribution characteristics of HFMD from 2008 to 2016 in Xinjiang as well as the global spatial autocorrelation and the incidence of HFMD in different regions. In addition, the factor detector module of GeoDetector were used to quantify the determinants of meteorological drivers of HFMD and the risk detector module of GeoDetector was used to detect the relative risks of HFMD under different meteorological elements. This study provides countermeasures and suggestions for further public health interventions.

## Materials and methods

### Study area

Xinjiang Uygur Autonomous Region is the largest provincial administrative region in China. In the region, the area is 1.66*106 km2 and the population is 24.87 million in 2018.

**Fig 1:**
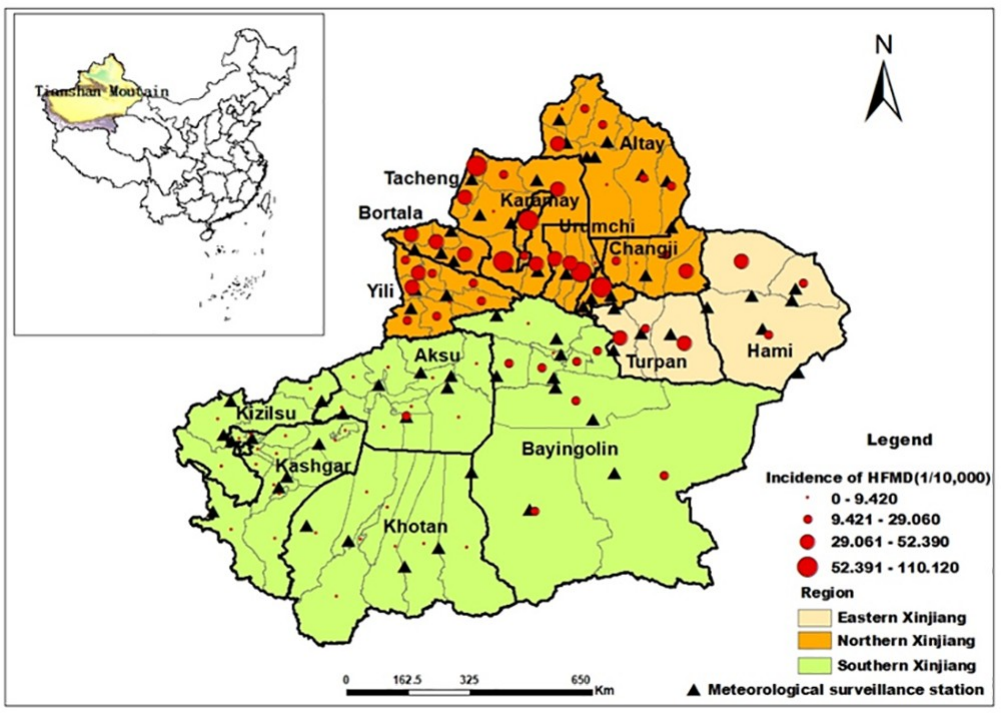
The study area (The original map is downloaded from the Gateway to Astronaut Photography of Earth Website (https://eol.jsc.nasa.gov/SearchPhotos/). Because the map downloaded from this website is free and open to scholars, our study does not need to supply a copyright permission).

Xinjiang is located in the geographical center of Eurasia (34.3°–49.5 °N, 73.5°–96.3 °E) and neighbors Russia, Kazakhstan, Kyrgyzstan, Tajikistan, Pakistan, Mongolia, India and Afghan from north to south. The mountains border Xinjiang on three sides and the Tianshan Mountains cuts across Xinjiang from east to west. As a typical arid and semi-arid area, Xinjiang has a temperate continental climate. The annual average air temperature ranges from 9 °C to 12 °C and the annual precipitations in Northern Xinjiang and Southern Xinjiang are respectively 210 mm and less than 100 mm, displaying an uneven spatial distribution pattern. The Tianshan Mountains has more precipitation, whereas Southern Xinjiang suffers the severe water stress. The dominant wind is northwest wind. Fig 1 shows the geographical location of Xinjiang.

### Data sources

The data of daily HFMD cases in Xinjiang from January 1, 2008 to December 31, 2016 were from China Information System for Disease Control and Prevention. The collected patient’s data include gender, age, living address, types of patients, the onset date of symptom, and confirmation time of symptom. This study was reviewed and approved by the Ethics Committee of the Xinjiang Center for Disease Control and Prevention, China. We aggregated the HFMD for each day at the county level (excluding the Xinjiang production and construction corps), not referring to patient’s privacy information. Meteorological data were obtained from the China Meteorological Data Sharing Service System [26], including daily average air temperature (TEM), daily average relative humidity (RHU), daily average barometric pressure (PRS), daily cumulative precipitation (PRE), daily average evaporation (EVP), daily average wind speed (WIN) and daily sunshine duration (SSD). Monthly average air temperature, relative humidity, barometric pressure, precipitation, evaporation, wind speed and sunshine duration were computed or aggregated from daily weather data. Monthly county-level meteorological variables data were collected from 66 meteorological surveillance stations in Xinjiang. The ordinary spatial Kriging interpolation in ArcGIS10.1 was used to interpolate meteorological data. The 66 meteorological surveillance stations are mapped in Fig 1.

### Methods

The authors assert that all procedures contributing to this work comply with the ethical standards of the relevant national and institutional committees.

#### GeoDetector

GeoDetector is a statistical method to detect the temporal-spatial heterogeneity. It is good at quantitatively express the spatial stratified heterogeneity of the research object by analyzing the differences and similarities of intra-layer and inter-layer variances (http://www.geodetector.cn/). It is composed of four modules: factor detector, interaction detector, risk detector and ecological detector. This tool has been widely used in land use, disease risk factor detection and other fields [27,28].

#### Factor detector

The q-statistic in GeoDetector [29] was used to quantify the spatiotemporal heterogeneity of HFMD and detect the interaction relationship between temporal and spatial effects in the study. In the study, GeoDetector was adopted to identify the risk factors that caused the temporal-spatial stratified heterogeneity of HFMD in Xinjiang from 2008 to 2016 from 7 candidate meteorological factors. The q-statistic is expressed in Eq (1) (The q-statistic for heterogeneity analysis regarding covariates was provided in the R Geodetector software):

$$q=1-\frac{1}{N\sigma^{2}}\sum_{h=1}^{L}N_{h}\sigma_{h}^{2}=1-\frac{SSW}{SST}\;SSW=\sum_{k=1}^{L}N_{k}\sigma_{k,}^{2}SST=N\sigma^{2},$$ (1)

where q denotes the explanatory power of a risk factor and quantifies the spatiotemporal stratified heterogeneity of the dependent variables such as HFMD risk (the value of q is strictly within [0, 1]); N is the number of all units, which can be divided into L strata; Stratum h is composed of *N*_*h*_ units; SSW and SST respectively indicate the sum of squares and the total sum of squares; *σ*^2^ and $\sigma_{h}^{2}$ are respectively the variance of all the units and the variance in Stratum h (h = 1,…,L).

#### Interaction detector

In order to detect the interactions between different risk factors X1 and X2, the interaction detector module of GeoDetector can reflect whether the interaction increases or decreases the explanatory power of the target variable Y. The relationship between the two factors can be divided into the following five categories [30]:

Weakening (nonlinear): q(X1∩X2) < Min(q(X1), q(X2))
Weakening (univariate): Min(q(X1), q(X2)) < q(X1∩X2) < Max(q(X1), q(X2))
Enhancement (bivariate): q(X1∩X2) > Max(q(X1), q(X2))
Independent: q(X1∩X2) = q(X1) + q(X2)
Enhancement (nonlinear): q(X1∩X2) > q(X1) + q(X2)

#### Risk detector

The spatial heterogeneity of impact factors has different effects on target variables in different regions. The t-statistic was used to determine whether there was a significant difference in the impact of meteorological factor on HFMD between two sub-regions, as expressed in Eq (2):

$$t_{{\bar{y}}_{h=1}-{\bar{y}}_{h=2}}=\frac{{\bar{Y}}_{h=1}-{\bar{Y}}_{h=2}}{{\left[\frac{Var({\bar{Y}}_{h=1})}{n_{h=1}}+\frac{Var({\bar{Y}}_{h=2})}{n_{h=2}}\right]}^{1/2}},$$ (2)

where ${\bar{Y}}_{h}$ represents the average value of attributes within sub-region h, such as the incidence of HFMD; *n*_*h*_ is the number of HFMD cases in sub-region h; Var is the variance.

#### Spatial autocorrelation analysis

Spatial autocorrelation analysis [31,32] involves global spatial autocorrelation and local spatial autocorrelation. In this study, Moran’s I of global spatial autocorrelation was used to describe the general spatial autocorrelation and the spatial distribution of HFMD cases in Xinjiang. LISA of local spatial autocorrelation reflects the specific cluster regions and cluster categories of HFMD cases in Xinjiang. Global spatial autocorrelation methods and local spatial autocorrelation methods were implemented in GeoDa V1.2.0 software (http://geoda.uiuc.edu/downloadin.php). The Moran’s I statistic is calculated according to Eq (3):

$$I=\left[n\sum_{i=1}^{n}\sum_{j=1}^{n}\omega_{ij}(X_{i}-\bar{X})(X_{j}-\bar{X})\right]/\left[\sum_{i=1}^{n}\sum_{j=1}^{n}\omega_{ij}\sum_{i=1}^{n}{\left(X_{i}-\bar{X}\right)}^{2}\right],$$ (3)

where *n* denotes the number of observed values; *X*_*i*_ represents the incidence rate in the region *i*; *X*_*j*_ represents the incidence rate in the region *j*; $\bar{X}$ indicates the average value; *ω*_*ij*_ is a spatial weight matrix of the systematic binomial distribution and represents neighboring relations between geographical units; n represents the total number of those units. In the study, the data were based on regions. If region *i* and region *j* are adjacent regions, *ω*_*ij*_ is equal to 1, otherwise *ω*_*ij*_ is equal to 0.

The range of Moran’s I value is [–1, 1] and the Moran scatter diagram represents the spatial agglomeration between a unit and its surrounding units. When I >0, there is a positive spatial autocorrelation between space units within the range. In other words, the difference in attribute values between adjacent space units is small. The two distribution patterns of attribute values, “low-low” clustering and “high-high” clustering, are presented. Moreover, the closer I value is to 1, the closer relationship between space units is and the smaller difference between attribute values is. When I <0, there is a negative spatial autocorrelation between spatial units within the range. In other words, there is a significant difference in the attribute values of adjacent spatial units. When the I value is closer to -1, the distribution pattern between spatial units is less concentrated and the difference in attribute values is more significant. When I = 0, there is no spatial autocorrelation between spatial units within the range and spatial variables present a random distribution pattern.

LISA was used to indicate the degree of correlation between a region unit and some attributes of an adjacent region unit. LISA cluster maps visualized the local spatial autocorrelation, which was classified into four types: high-high cluster (HH, which indicated that the highly clustered areas were surrounded by other highly clustered areas), low-low cluster (LL, which indicated that the lowly clustered areas were surrounded by lowly clustered areas), high-low cluster (HL), and low-high cluster (LH).

## Results

### Descriptive analysis

**Fig 2:**
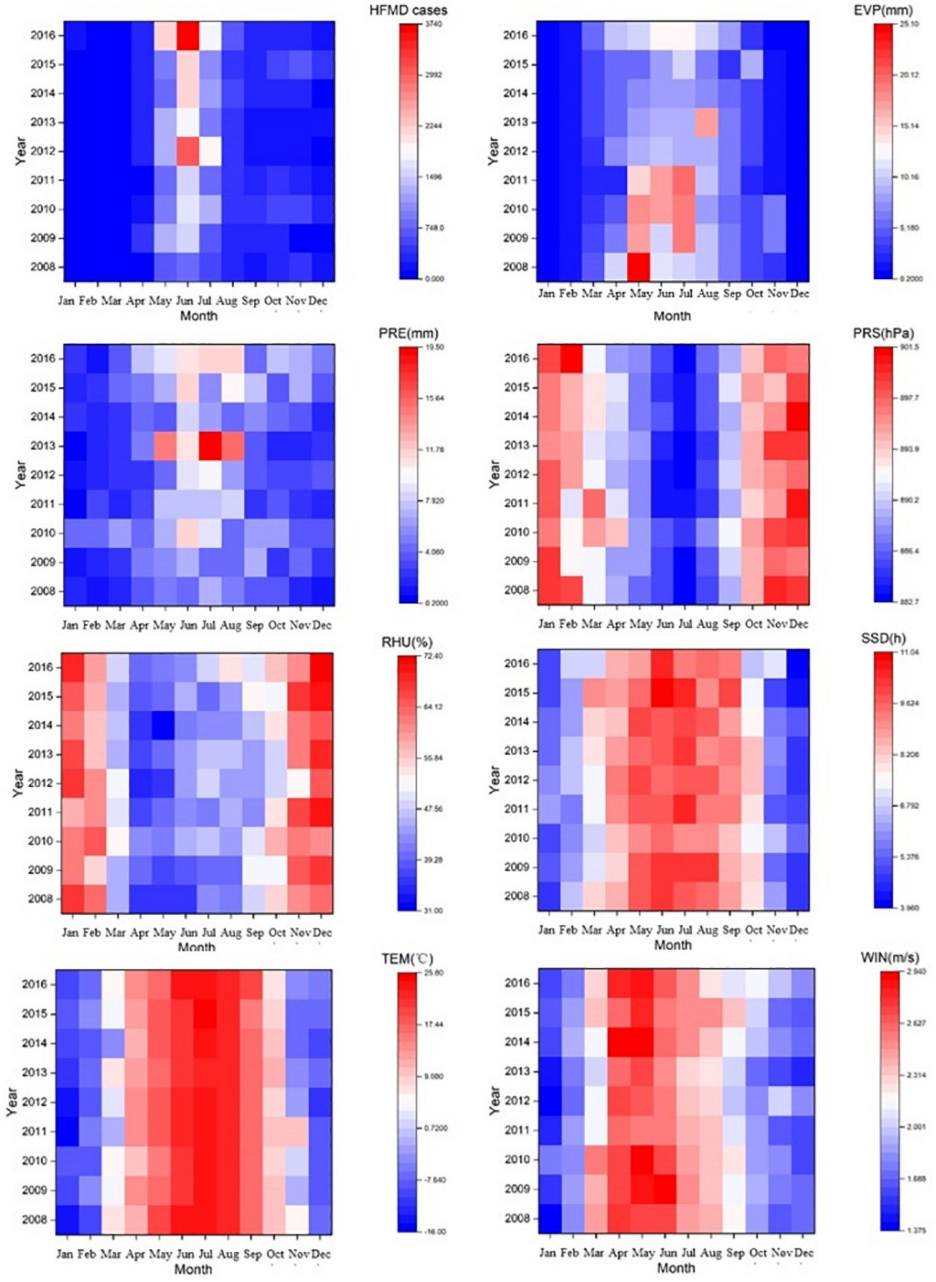
Heatmap of monthly data of HFMD cases and meteorological variables in Xinjiang, China from 2008 to 2016.

In total, 56,379 HFMD cases were reported from 2008 to 2016 in Xinjiang, with the daily average of 17.2 cases and the annual average incidence of 25.27/10,000. Fig 2 shows monthly distributions of HFMD cases and meteorological variables in Xinjiang from 2008 to 2016. The incidence of HFMD was significantly correlated with the changes of meteorological variables. Monthly HFMD distribution showed a distinct seasonal pattern over the period and HFMD cases typically occurred from May to July and peaked in June. The annual morbidity among males was about 1.5 times higher than that among females. Children aged below 5 were at the highest risk of HFMD. Most cases (86.2%) were dispersed children who did not go to kindergarten or school.

Table 1 illustrates the descriptive statistics of meteorological variables in the study period. The monthly average relative humidity, monthly average air temperature, monthly average precipitation, monthly average sunshine duration, monthly average wind speed, monthly average barometric pressure and average evaporation were 51.52% (32.35 ~ 70.69%), 8.82 °C (– 0.01 ~ 17.63 °C), 0.95mm (0.02~1.91 mm), 7.89 h (5.72 ~ 10.05 h), 2.53 m/s (0.84~ 4.21 m/s), 856.08 hPa (699.42~ 1012.74 hPa), and 3.68 mm (0.16 ~7.2 mm).

**Table 1:** Descriptive statistics of meteorological variables.

Covariates	Minimum	25% Percentile	Median	75% Percentile	Maximum
Average relative humidity (%)	32.35	41.64	51.52	61.11	70.69
Average air temperature (◦C)	0.01	4.42	8.82	13.23	17.63
Average precipitation (mm)	0.02	0.49	0.95	1.42	1.91
Average sunshine duration (hour)	5.72	6.8	7.89	8.97	10.05
Average wind speed (m/s)	0.84	1.68	2.53	3.37	4.21
Average barometric pressure (hPa)	699.42	777.75	856.08	934.41	1012.74
Average evaporation (mm)	0.16	1.92	3.68	5.44	7.2

#### Factor detector analysis

As shown in Table 2, the determinant power of the average relative humidity is correlated with the incidence of HFMD (q = 0.30), indicating that the average relative humidity mainly explains the spatial heterogeneity of the incidence of HFMD. Precipitation, barometric pressure, temperature and sunshine duration were also correlated with the incidence of HFMD in Xinjiang and had the explanatory power q of 0.29, 0.29, 0.21 and 0.20, respectively. The study revealed that humidity, precipitation and barometric pressure were three main factors influencing the transmission of HFMD in Xinjiang.

**Table 2:** Explanatory power of each factor on the incidence of HFMD in Xinjiang.

	EVP	PRE	PRS	RHU	SSD	TEM	WIN
*q* statistic	0.11	0.29	0.29	0.30	0.20	0.21	0.07
p value	0.000	0.000	0.000	0.000	0.05	0.000	0.05

#### Interaction detector

The interaction of any two meteorological factors had the greater explanatory power than any single meteorological factor. The interaction between any two meteorological factors presented the effect of “nonlinear enhancement” or “bivariate enhancement” on the incidence of HFMD. The q-statistic of the interactive effect of average barometric pressure and average relative humidity (0.5) was significantly higher than that of average relative humidity (0.3) and average barometric pressure (0.29), indicating that the bivariate enhanced interaction between relative humidity and barometric pressure was significant (Table 3). The interactive effect between precipitation and average relative humidity increased to 0.39 on HFMD. The coupled impact between average relative humidity (q = 0.3) and average wind speed (q = 0.07) played an important role in the incidence of HFMD, with an explanatory power of 0.43 (Table 3), indicating that high average relative humidity and high average wind speed were correlated with a high incidence of HFMD. The interaction among these meteorological factors could effectively explain why two meteorological factors were the most sensitive to the incidence of HFMD.

**Table 3:** Values of *q* for the interactions between pairs of factors on the incidence of HFMD.

Covariates	EVP	PRE	PRS	RHU	SSD	TEM	WIN
EVP	0.11						
PRE	0.34EB	0.29					
PRS	0.35EB	0.37EB	0.29				
RHU	0.35EB	0.39EB	0.50EB	0.30			
SSD	0.21E	0.33EB	0.40EB	0.38EB	0.20		
TEM	0.34EN	0.33EB	0.41EB	0.37EB	0.34EB	0.21	
WIN	0.25EN	0.36ID	0.36ID	0.43EN	0.35EN	0.32EN	0.07

Note: EN: Enhancement (nonlinear), EB: Enhancement (bivariate), ID: Independent, E: Enhancement

#### Risk detector analysis

**Fig 3:**
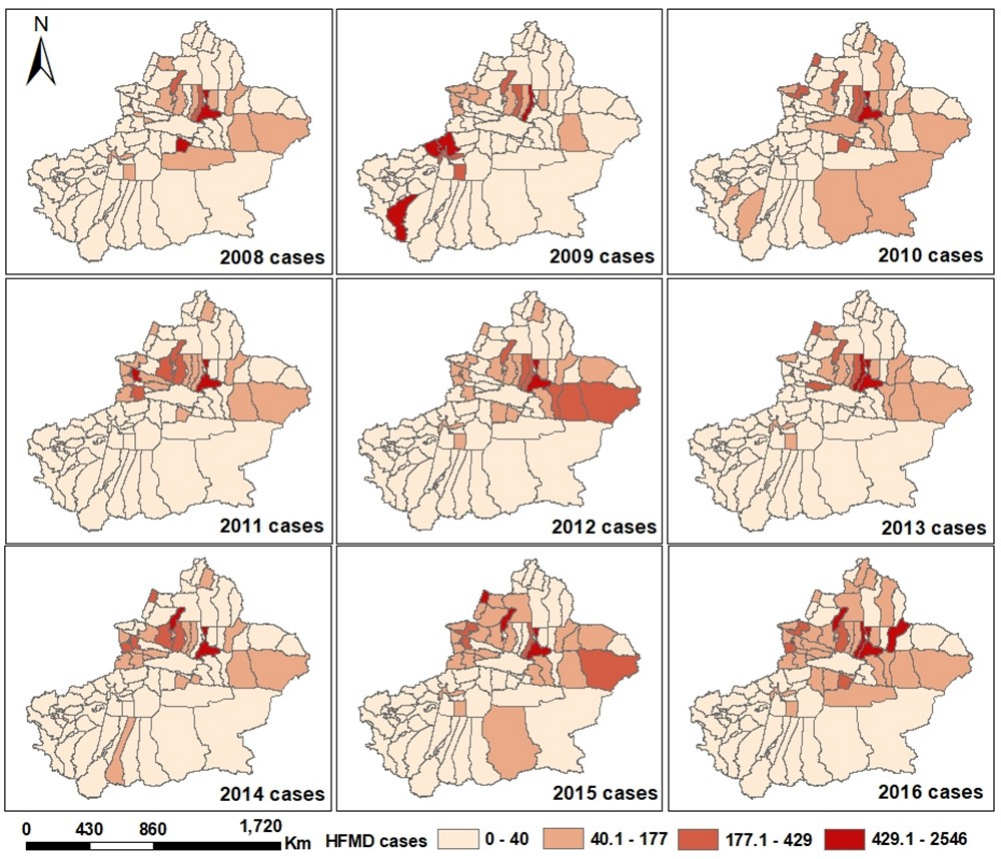
Spatial-temporal distributions of HFMD cases in Xinjiang. The original map is downloaded from The Gateway to Astronaut Photography of Earth Website (https://eol.jsc.nasa.gov/SearchPhotos/). Because the map downloaded from this website is free and open to scholars, our study does not need to supply a copyright permission.

Fig 3 shows the spatial distribution of HFMD cases in Xinjiang from 2008 to 2016. The number of HFMD cases is divided into four grades by natural breaks in ArcGIS. A deeper red color indicates a larger number of HFMD cases. Most of them were concentrated in Northern Xinjiang.

**Fig 4:**
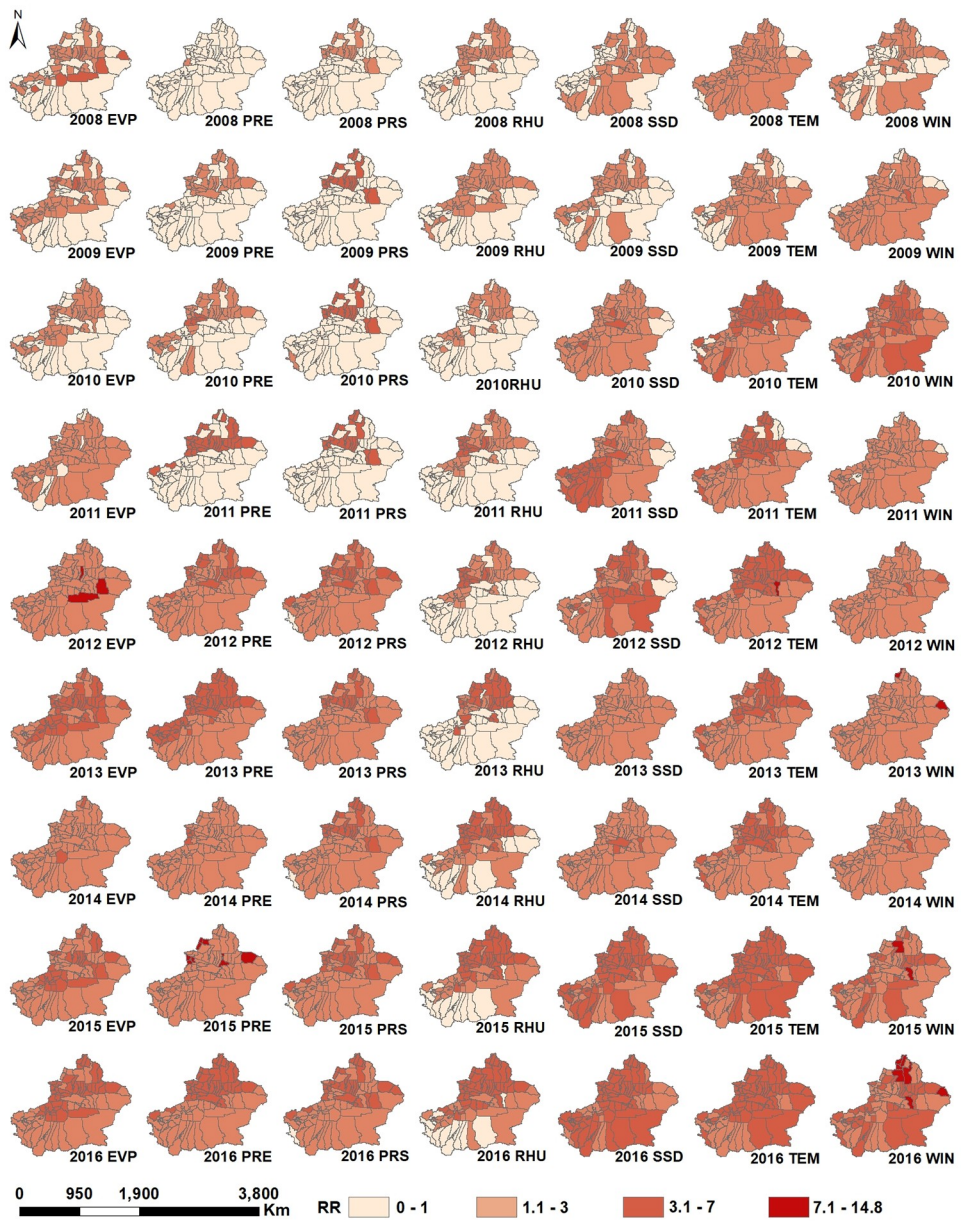
Relative risk (RR) of HFMD under the influences of meteorological factors. (The original map is downloaded from the Gateway to Astronaut Photography of Earth Website (https://eol.jsc.nasa.gov/SearchPhotos/). Because the map downloaded from this website is free and open to scholars, our stud does not need to supply a copyright permission).

The risk detection results obtained with the GeoDetector are discussed below. Fig 4 shows the relative risk (RR) of HFMD with different meteorological factors from 2008 to 2016 in Xinjiang. Actually, the spatial distribution of RR was not the same in different years. The spatial RRs in counties in Northern Xinjiang were higher than those in the counties in Southern Xinjiang, implying that these counties had a relatively higher HFMD risk. Conversely, the counties in Southern Xinjiang generally had the lower RRs. The spatial distribution difference was consistent with the results shown in Fig 3. Northern Xinjiang had a higher average relative humidity, suitable temperature and precipitation, thus resulting in a higher RR of HFMD. The southern regions are affected by the Taklimakan desert, high temperature, and low relative humidity, precipitation and barometric pressure, so the risk of HFMD was relatively low. We found the lowest RR of HFMD in Khotan during the study period. As indicated by the RR of HFMD, Urumqi, Tacheng Prefecture, Changji Prefecture and Ili Kazak Autonomous Prefecture are the high RR areas of HFMD in Northern Xinjiang. It may be ascribed to the higher average relative humidity and sufficient precipitation in the above areas. These meteorological conditions are suitable for the transmission of the HFMD virus.

**Fig 5:**
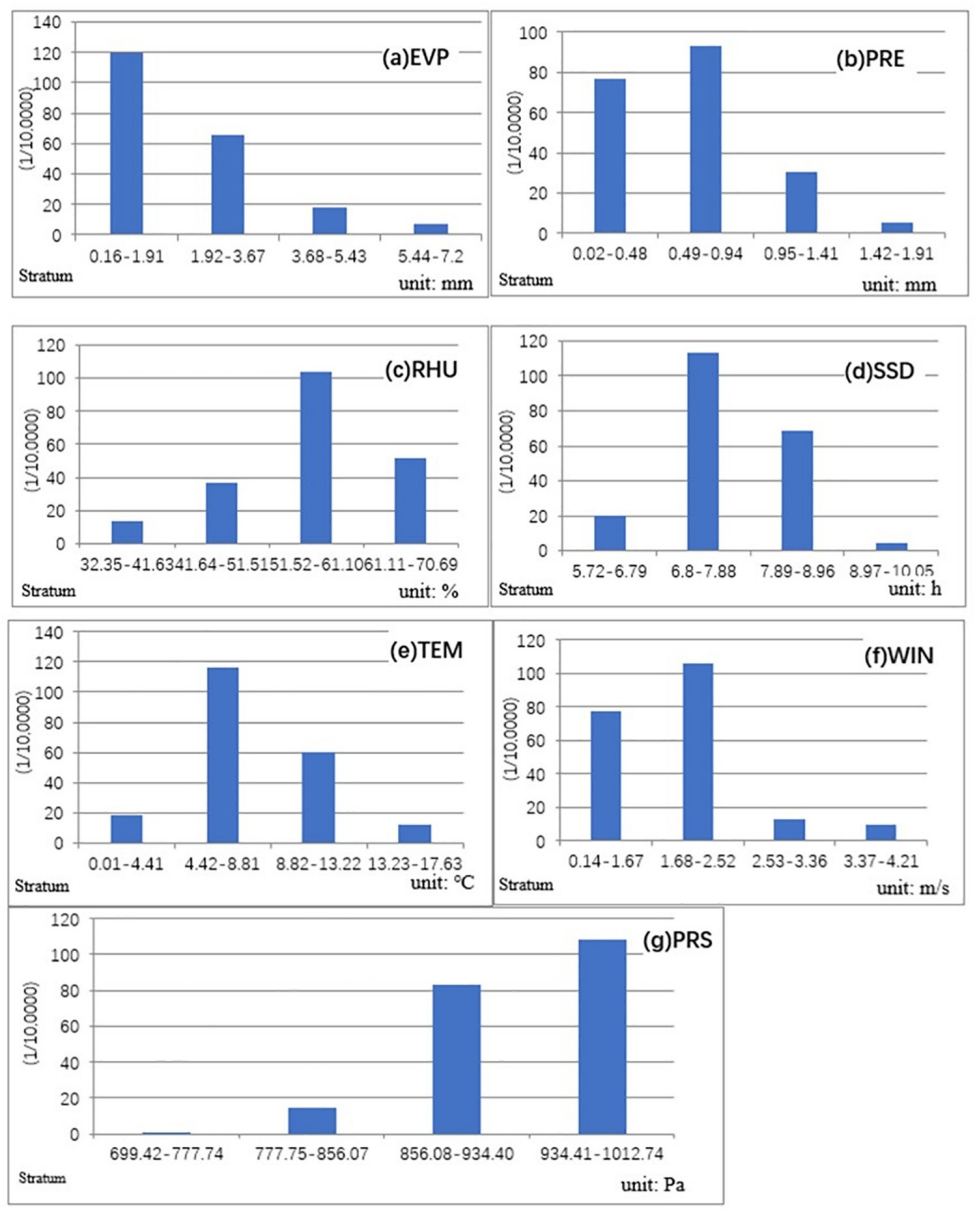
Average incidence rates of HFMD calculated with different risk factor strata. a. Evaporation; b. Precipitation; c. Relative humidity; d. Sunshine duration; e. Temperature; f. Wind speed; g. Pressure. (Vertical axis indicates the incidence of HFMD; horizontal axis indicates the group of meteorological factors).

When monthly average precipitation exceeded 0.94 mm, the incidence of HFMD decreased (Fig 5b). Monthly average precipitation range of 0.02–0.94 mm accounted for 82.5% of the total incidence of HFMD in Xinjiang. There was an inverted V-shaped correlation between monthly average air temperature and HFMD. A similar pattern was observed from the correlation between HFMD and other four meteorological factors (monthly average relative humidity, monthly average sunshine duration, monthly average precipitation, and monthly average wind speed) (Fig 5c, 5d and 5f). When monthly average relative humidity was 51.52% to 70.69%, the incidence of HFMD was higher than that in other relative humidity intervals (Fig 5c). The sensitive monthly average sunshine duration for HFMD was 6.8 to 8.96 hours (Fig 5d). When monthly average air temperature was 8.81 °C, the incidence of HFMD peaked. The incidence of HFMD was higher in the temperature range from 4.22 °C to 13.22 °C (Fig 5e). With the increase in monthly average relative humidity and monthly average sunshine duration, the incidence of HFMD increased, peaked under the conditions of monthly average relative humidity of 61.1% and monthly average sunshine duration of 7.78 hours and then decreased. Risk detector value revealed a logarithmic relationship between monthly average evaporation and the incidence of HFMD and an exponential relationship between monthly average barometric pressure and the incidence of HFMD (Fig 5a and 5g). The incidence of HFMD was the highest when monthly average wind speed was less than 2.52 m/s (Fig 5f). It should be noted that when a single meteorological factor mentioned above reached the range corresponding to a high incidence of HFMD, it did not necessarily mean the outbreak of HFMD, which also required the comprehensive action of other meteorological factors.

### Spatial autocorrelation of the incidence of HFMD

Moran’s I value was calculated through the global spatial autocorrelation analysis. From 2008 to 2016, the Moran’s I value of HFMD in Xinjiang (Table 4) fluctuated in the range from -0.135 to 0.202 (P < 0.05), indicating the spatial dependency of HFMD in 2008, 2010, 2012, 2014 and 2015. Moran’s I value in 2009 was -0.135, indicating that there was a negative spatial autocorrelation of HFMD in Xinjiang. Moran’s I values in 2011 and 2016 were respectively -0.066 and -0.00018, indicating that the incidence of HFMD in Xinjiang presented a random distribution pattern. Bayingolin Mongol Autonomous Prefecture showed the high-high spatial autocorrelation with the incidence of HFMD, whereas Kashgar, Hotan, Aksu and Kizilsu Kirghiz Autonomous Prefecture showed the low-low spatial autocorrelation with the incidence of HFMD in 2008 and 2010. From 2011 to 2016, Urumqi always showed the high-high spatial autocorrelation with the incidence of HFMD.

**Table 4:** Results of the spatial autocorrelation test on HFMD cases in Xinjiang from 2008 to 2016.

Years	2008	2009	2010	2011	2012	2013	2014	2015	2016
Moran’s I	0.144	-0.135	0.202	-0.066	0.120	0.056	0.134	0.146	-0.001
Z-score	1.904	-0.647	2.832	0.042	1.933	1.181	2.092	2.146	0.625
P values	*P*<0.05	*P*<0.05	*P*<0.05	*P*<0.05	*P*<0.05	*P*<0.05	*P*<0.05	*P*<0.05	*P*<0.05

**Fig 6:**
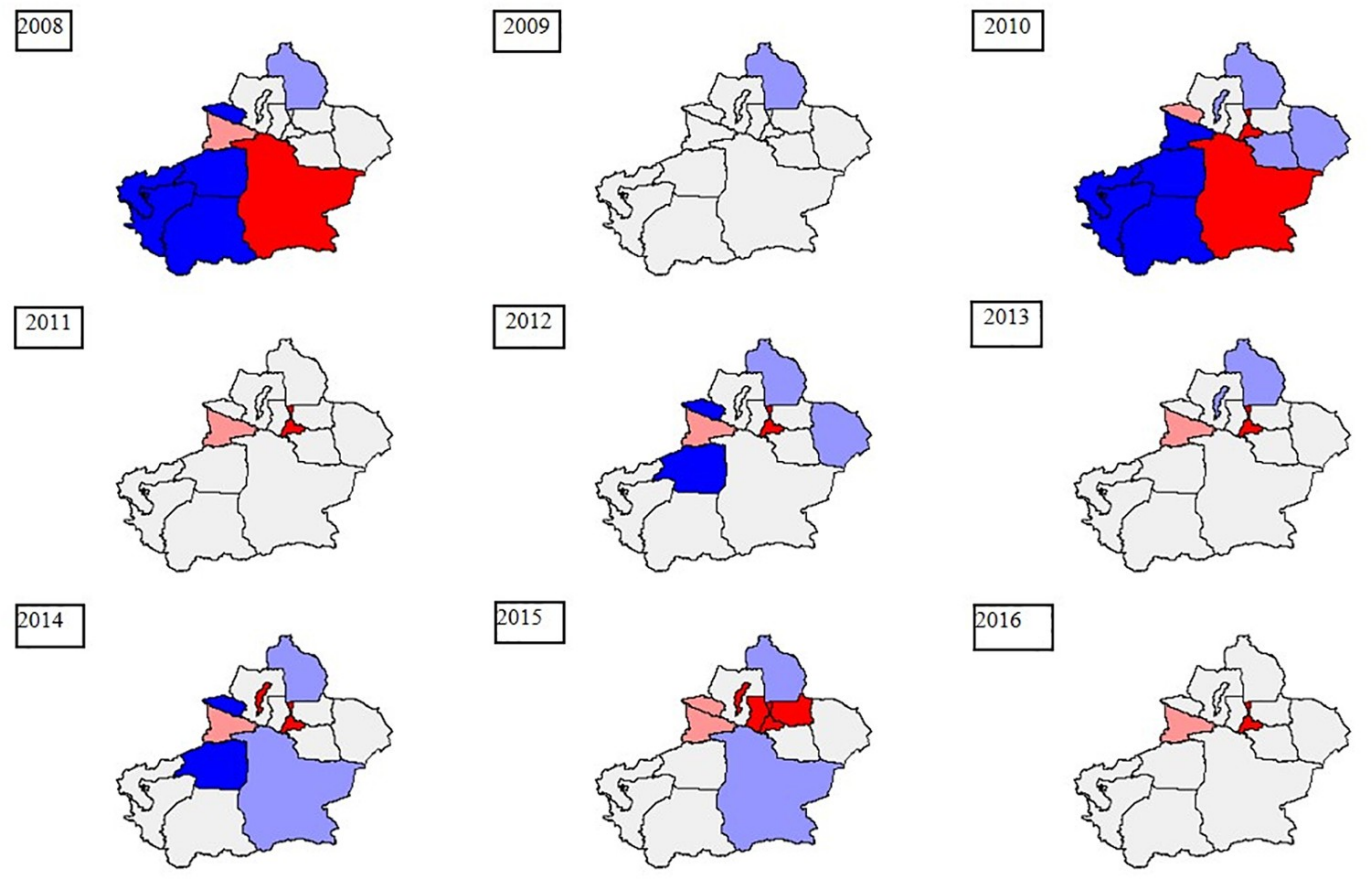
Results of local spatial autocorrelation analysis in Xinjiang from 2008 to 2016. (The original map is downloaded from the Gateway to Astronaut Photography of Earth Website (https://eol.jsc.nasa.gov/SearchPhotos/). Because the map downloaded from this website is free and open to scholars, our study does not need to supply a copyright permission).

In this study, we presented an objective fact of spatial autocorrelation of HFMD in Xinjiang (Fig 6).

In 2008, 2010, 2012, 2014 and 2015, H-H (Bayingol Urumchi Changji Karamay) and L-L (Kashgar, Hotan, Aksu and Kizilsu) spatial autocorrelation dominated, indicating the spatial dependency on the occurrence of HFMD.

In 2011 and 2016, the spatial autocorrelation showed the same characteristics. Only two prefectures (Urumchi and Ili) showed H-H and L-H spatial correlations with other prefectures and the other 12 prefectures were all grey. In 2013, three prefectures showed H-L and L-H spatial correlation with other prefectures. The value of Moran’s I was the closest to 0 (-0.066, 0.056, and -0.001), indicating that the incidence of HFMD in Xinjiang presented a random distribution pattern.

In 2009, only one prefecture showed H-L (Altay) spatial autocorrelation with other prefectures. The value of Moran’s I was -0.135, indicating that Altay had a negative spatial autocorrelation with the incidence of HFMD in Xinjiang.

## Discussion

In recent years, HFMD has become a significant health problem [33,34]. Moreover, in the areas with different climate types, the HFMD risk was significantly different. The correlation between the incidence of HFMD and climatic factors has been extensively explored [6,15]. The relationships between the incidence of HFMD and various meteorological factors in Xinjiang were interpreted with daily HFMD surveillance data for the first time in the study. The study on HFMD in such arid and semi-arid areas would further reveal the transmission mechanism of HFMD under different climate conditions.

Previous studies indicated that the response of the incidence of HFMD to climate change differed. In temperate regions, HFMD outbreaks usually occurred in summer or early fall [5] since temperature rise and precipitation increase occurred later than those in tropical regions. Therefore, the incidence of HFMD in temperate regions was different from that in tropical and subtropical regions [19,34]. For example, in Vietnam, the incidence of HFMD peaked in April, whereas it peaked in June in Gansu, China [35]. The climate types of the two places mentioned above differed markedly. The study on the response of meteorological factors to HFMD obtained some different results from the previous studies. For instance, in Guangdong in South China, a nonlinear relationship between temperature and the incidence of HFMD was reported [13]. The positive correlation between temperature increases and the incidence of HFMD was corroborated in other studies. For instance, in Japan, with the increase in temperature, the incidences of Herpangina & HFMD increased. In a warm environment, the transmission of HFMD was enhanced, but cold and hot climates limited HFMD transmission [5,6,24]. Similarly, in Ningxia in the upper reaches of the Yellow River in Western China, average air temperature, relative humidity, and wind speed played significant roles in the spatial-temporal distributions of HFMD risk [14].

In our research, the relationship between monthly average air temperature and HFMD relative risk represented an inverted V-shaped pattern. A similar pattern was observed from the correlations between HFMD and other four meteorological factors (monthly average relative humidity, monthly average precipitation, monthly average sunshine duration, and monthly average wind speed). The relationships between meteorological factors and the risk of HFMD in Xinjiang were non-linear relationships. Furthermore, in Guangdong, China, where the incidence of HFMD was high, the risk of HFMD was significantly correlated with monthly average relative humidity, monthly average air temperature, and monthly average rainfall. In this study, from the results of GeoDetector, an increase in the incidence of HFMD was found to be correlated with monthly average relative humidity, monthly average precipitation, monthly average barometric pressure and monthly average air temperature. The relative humidity had the strongest explanatory power. Previous studies in Zhejiang [36], Guangdong [37,38] and other regions showed that temperature has the strongest influence on HFMD among meteorological factors. The studies on the relationship between meteorological factors and HFMD in Henan [11] and Shandong [15] showed that relative humidity had a stronger effect on HFMD than temperature. In Ningxia [14], average air temperature and relative humidity were the dominant factors in the spatial-temporal distribution of HFMD. The inconsistency of the research results was ascribed to different climate types in study areas. Xinjiang is located in an arid and semi-arid climate zone with average annual relative humidity of 50.5%, which is lower than that in the central and eastern regions of China. However, in the central and eastern regions of China, the annual change of relative humidity is much less significant than that of temperature. Therefore, the daily incidence of HFMD was sensitive to the change of temperature in the central and eastern regions of China. On the contrary, in arid and semi-arid areas, the change of relative humidity as sensitive to the incidence of HFMD. The differences in climate and latitude between Southern and Northern Xinjiang and the arid and semi-arid geographical environments were partially responsible for the distribution difference of HFMD in Xinjiang from other temperate continental climatic zones. These correlations highlight climate-related health issues and shall be considered in the development of accurate spatiotemporal prevention measures of HFMD in Xinjiang, China. In addition to relative humidity, the synergistic effects of other meteorological factors also affected the daily incidence of HFMD in Xinjiang. Climate conditions might also have a threshold effect [39]. It should be noted that the thresholds of meteorological factors corresponding to the incidence of HFMD in Xinjiang were significantly different from those in other study areas. A study conducted in mainland China showed that relative humidity of 80.59% to 82.55% would lead to a higher risk of HFMD [40]. However, monthly average relative humidity of 51.52% to 61.10% was the threshold for the high incidence of HFMD in Xinjiang. In our previous study [41], we found the correlation between temperature, precipitation and the incidence of HFMD in Xinjiang China. The risk detector value presented a logarithmic relationship between monthly average evaporation and HFMD and an exponential relationship between monthly average barometric pressure and HFMD. The spatial-temporal heterogeneity of HFMD in Xinjiang was reported by us for the first time.

The above relationships between HFMD and meteorological factors might be ascribed to the stability of the HFMD enterovirus in the external environment, such as humidity and temperature. Moderate monthly cumulative precipitation could maintain the prevalence of HFMD because precipitation might affect water sanitation, promote the attachment of HFMD virus, increase the risk of exposure, and facilitate the spread of HFMD [1,12].

In order to further reveal the relationships between meteorological factors and the incidence of HFMD, in this study, the spatial and temporal heterogeneity and their interactive effects on the incidence HFMD in Xinjiang were explored with GeoDetector and the spatial pattern of HFMD in Xinjiang was investigated with the spatial autocorrelation analysis method. The incidence of HFMD showed significant regional differences, displaying a dynamic spatial-temporal distribution. HFMD cases were mainly concentrated in Northern Xinjiang. The incidence of HFMD in Southern Xinjiang might be ascribed to the precipitation stress. The incidence of HFMD in Urumqi, Changji Prefecture, Tacheng Prefecture and other areas in Northern Xinjiang was worthy of in-depth investigation. We believed that in these focal areas meteorological factors were the predisposing factors of HFMD and that the well-developed economy, fast highway system, and large heterogeneous migrant population also increased the HFMD risk [17].

The global spatial autocorrelation analysis results demonstrated that the area with the high incidence of HFMD was different from the high spatial autocorrelation area of HFMD in Xinjiang. Urumqi always showed the high spatial autocorrelation of the incidence of HFMD because the incidence of HFMD in Urumqi remained high over the years and adjacent areas also had a high incidence of HFMD. Multiple regions showed the low spatial autocorrelation because the incidence of HFMD had inter-annual variations in several regions and the incidence of HFMD in adjacent areas was low, such as Aksu and Ili Kazak Autonomous Prefecture.

The study has some limitations. First, there were only 66 meteorological surveillance stations in Xinjiang and ordinary Kriging interpolation results might not cover all variations of the meteorological variables at the county level. However, it was more accurate than the result obtained with the same meteorological data in all counties in each city. Second, we estimated spatio-temporal variations in HFMD at the scales of counties and months and we did not include the factors at an individual and pathogenic levels, such as personal hygiene, educational background, income of children’s parents, living conditions, and the composition of major pathogens, and social environmental factors such as the population density, health facilities and services. The potential impacts of these factors should be considered in the future studies.

## Conclusions

The study indicated that the spatial-temporal distribution of HFMD risk in Xinjiang, China was non-homogeneous. The counties with the higher relative risk were mainly located in Northern Xinjiang. Meteorological factors, such as monthly average relative humidity, monthly average precipitation, monthly average barometric pressure and monthly average air temperature, were the driving factors of HFMD in Xinjiang. Both the areas with high relative risks and the areas with the high spatial correlation of HFMD were located in Northern Xinjiang. Therefore, in the season with a high incidence of HFMD and in areas with a high relative risk, we can reduce environmental exposure and contact transmission in order to decrease the spread of HFMD.
